# ACPSEM position paper on ROMP scope of practice and staffing levels for magnetic resonance linear accelerators

**DOI:** 10.1007/s13246-023-01253-4

**Published:** 2023-04-11

**Authors:** Linda Marsh, Kym Rykers, Matthew Sobolewski, the Magnetic Resonance Imaging Linac Working Group (MRILWG) of the ACPSEM

**Affiliations:** 1 Townsville Cancer Center, Douglas, QLD Australia; 2 Austin Health, Heidelberg, VIC Australia; 3 Cancer Care Associates, Miranda, NSW Australia

## Abstract

The purpose of this position paper is to outline the ACPSEM recommendations on Medical Physicist scope of practice and staffing levels, as they relate to the use of dedicated MRI-Linacs in the treatment of patients. A core function of Medical Physicists is to safely implement changes in medical practice via the introduction of new technology and to ensure high quality radiation oncology services are provided to patients. Determining the feasibility of MRI-Linacs in any existing setting, or in establishing a new site, mandates the knowledge and services of Radiation Oncology Medical Physicists (ROMPs) as the Qualified Experts within this setting. ROMPs are key members of the multi-disciplinary team which will be required to steer the successful establishment of MRI Linac infrastructure within departments. To support efficient implementation, ROMPs must be embedded in the process from the start, including any feasibility study, initiation of the project, and development of the business case. ROMPs must be retained throughout all stages of acquisition, service development, and ongoing clinical use and expansion. The number of MRI-Linacs in Australia and New Zealand is growing. This expansion is occurring in parallel with rapid technological evolution, expanding tumour stream applications, and increasing consumer uptake. Growth and applications of MRI-Linac therapy will continue to occur beyond current known horizons, via development on the MR-Linac platform itself and through the migration of learning from this platform to conventional Linacs (known horizons for example include the use of daily, online image guided adaptive radiotherapy and MRI data informing decision making for planning and treatment before and throughout treatment courses). Clinical use, research and development will be a significant component of expanding patient access to MRI-Linac treatment and there will be an ongoing need to attract and retain ROMPs to initially establish services and in particular to drive service development and delivery for the life of the Linacs. MRI and Linac technologies mean it is necessary to perform a specialized workforce assessment for these devices, distinct from those employed for conventional Linacs and associated services. MRI-Linacs are complex, have a heightened risk profile compared to standard Linacs, and are unique in their treatment of patients. Accordingly, the workforce needs for MRI-Linacs are greater than for standard Linacs. To ensure safe and high-quality Radiation Oncology patient services are provided, it is recommended that staffing levels should be based on the 2021 ACPSEM Australian Radiation Workforce model and calculator using the MRI-Linac specific ROMP workforce modelling guidelines outlined in this paper. The ACPSEM workforce model and calculator are closely aligned with other Australian/New Zealand and international benchmarks.

**Supplementary Information:**

The online version contains supplementary material available at 10.1007/s13246-023-01253-4.

## Medical physicist role related to MRI-Linacs in the clinical environment

The Medical Physicist workforce in the Australian and New Zealand health care context is comprised of specialty groups of radiation oncology, radiology, nuclear medicine, and radiopharmaceutical scientists. Radiation oncology medical physicists (ROMPs) are embedded in oncology departments and uniquely qualified to provide scientific and technical leadership to the oncology MRI-Linac team. Radiology medical physicists qualified in diagnostic medical imaging and with MRI expertise (MRI physicists) would ideally be available to the radiation oncology service. They would augment service development and clinical safety in the role of MRI Safety Advisor in line with RANZCR MRI Safety Guidelines [1].

The role of ROMPs in providing ongoing high-quality patient care in a radiotherapy setting is both obvious and subtle [2]. Numerous reviews in Australia and overseas have consistently highlighted the need for appropriate Medical Physicist staffing to mitigate risk and avoid large cohorts of mistreatment and sentinel events [3]. The ability to deliver safe, high-quality radiotherapy will be compromised by inadequate staffing levels, particularly in an environment where new technology and techniques are being rapidly developed and introduced [2]. It is recommended [4] to engage a scientist to provide high-level advice on the engineering, scientific and administrative aspects of the safe clinical use of MRI devices and in the Radiation Oncology environment the appropriate scientist is a ROMP. Aligned with RANZCR recommendations, the European Federation of Medical Physics (EFOMP) also recommends the provision of an MRI Safety Expert to provide “high-level advice on the engineering, scientific and administrative aspects of the safe clinical use of MRI devices” [5].

The ACPSEM position paper on the Safety of Magnetic Resonance Linear Accelerators [6] describes the roles necessary for safety. ROMPs have safety expertise, already routinely fulfilling the role of Radiation Safety Officer (RSO), this should be extended to the unique environment of the MRL with appropriate accreditation. ROMPs are most suited to roles offering technical advice such as MR Safety Expert (MRSE)/MR Safety Advisor and could also fulfill the MR Safety Officer (MRSO) role. MRSO/MRSE levels of competence can be achieved via professional credentialing and coursework combined with customary CPD activities. This competence should be established as early as possible in the project.

Recruitment of ROMP expertise early in the project, well before the installation of the MRI-Linac, is recommended. Determining the feasibility of MRI-Linacs in any existing setting, or in establishing a new site, mandates the knowledge and services of Radiation Oncology Medical Physicists (ROMPs) as the Qualified Experts within this setting [7]. ROMPs are key members of the multi-disciplinary team [8] which will be required to steer the successful establishment of MRI Linac infrastructure within departments. ROMP involvement in the building phases; procurement; IT and Electronic Medical Record infrastructure; MRI equipment installation; commissioning; clinical release, ongoing service development and research, including clinical trials, will enhance the establishment of an efficient, safe, and risk-aware service. Decisions made from project initiation to clinical use and beyond will impact existing patient workflows, installation options, MRI specific patient flows, MRI and Radiation Safety and planning for upgrades or replacements in the future. Retaining ROMP experience through all steps will ensure a high degree of technical and clinical expertise is in place throughout key decision-making moments of service development which in turn will support economic delivery of the service.

## Radiation oncology medical physicists’ strategic value

ROMPs bring strategic value and expertise across the following aspects of the use of MRI-Linacs in radiotherapy including but not limited to the items in Tables 1, 2, 3, 4 and 5 available as supplementary material and elaborated subsequently.

## Recommended staffing

ROMPs are crucial members of the radiation oncology service. ROMPs should be employed from the beginning of service planning and development. Risks with radiation and magnetic resonance imaging devices should not be underestimated and they are risks that persist for the duration of the life of the devices. As demonstrated in the scope of practice presented, ROMPs are highly skilled professionals tasked with a wide array of critical responsibilities necessary to facilitate the implementation, development, and management of an optimal MRI-Linac service.

The ACPSEM Radiation Oncology Medical Physics Workforce Model [9] guides appropriate staffing levels for registered ROMPs in Australia and New Zealand. The model and accompanying calculator are closely aligned with other Australian/New Zealand [10, 11] and international [12, 13]  benchmarks. MRI-Linacs are included within the scope of this activity-based model, and registered ROMP FTE estimates for MRI-Linac facilities may be derived. Complete information which informs these parameters may not be known in the development phase of new MRI-Linac facilities. In these circumstances, users of the model are advised to apply carefully considered estimates to ensure facilities are appropriately staffed to support optimal and safe service delivery. It is the recommendation of the ACPSEM that staffing levels for MRI-Linac facilities should be set according to the judicious application of the ACPSEM ROMP Workforce (ARW) Model calculator [14], with particular care taken when estimating the ROMP time per MRI-Linac patient.

When utilising the ARW Model calculator, the following guidelines are recommended:

1. Utilising an appropriately balanced ROMP activity work profile
  Standard Australia-New Zealand radiation oncology sites on average utilise 68% of the available ROMP hours to complete activities that are not patient or equipment QA activities. These includes activities that fall into a variety of categories including quality and safety, clinical and service development, and education.The remaining ROMP time is then allocated to routine patient and equipment QA.
  Maintaining a high percentage time allocation to non-routine QA tasks ensures the broader needs of the department are met. While at present evidence suggests that MRI-Linac treatment programs within the region, have a higher percentage of ROMP hours dedicated to patient specific QA activities, it is reasonable to expect that the average ROMP work profile will vary over time. This should be accounted for when inputting data into the ARW Model calculator which may be re-evaluated over the lifetime of the service.
2. Ensure the estimate for ROMP time per MRI Linac patient responsibly supports the intended clinical practice.
  International evidence of the operating model for new and established MRI-Linac treatment programs highlights that ROMP hours are more heavily invested in treatment decision making and planning at the treatment console as well as undertaking increased patient specific QA activities arising from the adaptive treatment approach of MRI-Linacs, than is routinely found with standard linacs.
  Median ROMP time per MRI patient will vary across services due to local operational models, tumour stream choice and prescription complexity and is likely to change with time as practices evolve. When users of the ARW Model calculator are determining values, they must combine all elements of ROMP time provided for the patient treatment quality assurance including treatment planning support, reference and fraction plan quality assurance, and attendance at treatment, utilising a weighted average of the different complexity level treatments provided.
3. Accurately determine the clinical capacity and likely throughput of the MRI-Linac
  Based upon overseas clinical evidence and local data, patient treatment fractions treated per day are lower than on conventional linacs. Considering that Australia-New Zealand radiation oncology sites typically operate 250 days per annum and that the expected number of fractions treated per day may average 10 for existing services, a reasonable capacity estimate of the MRI-Linac would be approximately 250 patients per annum with 10 fractions per treatment course average. The figures provided in this example are intended as a guide only and each department must determine their own capacity estimate. The use of average fractions per patient is also more nuanced with MRI-Linacs than standard linacs. Using this capacity estimate as an upper bound alongside a service growth plan, a reasonable figure for MRI-Linac patients per annum can be determined.
4. Fully account for all equipment related to the MRI-Linac service
  Equipment required to operate an MRI-Linac service extends far beyond the MRI-Linac itself and includes a wide variety of systems and hardware to ensure quality service delivery. The support of all these items must be adequately accounted for by ensuring they are included in the equipment section of the ARW Model calculator.

From the ROMPs appointed, one could fulfil the role of MRSO, and ideally, one member of the ROMP workforce should be educated to the MRSE level. These roles and responsibilities, are elaborated in the MRILWG safety recommendations [6].

## ROMP strategic value and scope of practice elaboration

### Service development & commissioning

#### Service scoping and design, site planning and configuration

Site and service planning is extensive for the MRI-Linac. The ability to treat patients successfully, safely, and efficiently using an MRI-Linac requires health services to audit and reimagine existing practices and building use to support MRI in Radiation Oncology.

Site planning and configuration are intrinsically linked with service scope and design. Critical to service scoping and design is the configuration of the physical environment, inclusive of MRI safety considerations. ROMPs are responsible for radiation safety and are the subject matter experts in radiation safety covering bunker design and retrofit; radiation shielding, protection of members of the public, patients and staff, and radiation surveying of radiation sources to ensure design goals are met. ROMPs also provide advice on how to mitigate variations and should work closely with the design teams to forestall any architectural or building deviations that would compromise radiation safety; this advice can save substantial time and money during a project. Furthermore, ROMPs are placed to provide advice on how departments operate when treating patients; describe typical patient flows and horizon scan to plan for machine replacement in future years.

Ongoing oversight, site inspection and review of scoping variations throughout the phases of the initial and capital work by Medical Physicists will also allow for early detection of any building omissions or oversights which will ensure safety for staff and patients and will save time and money for the health service.

#### Technical and clinical commissioning

Working closely with the vendor the ROMPs take responsibility on behalf of the health service to ensure the MRI-Linac is installed appropriately, that it meets specifications of the purchase agreement and as soon as radiation can be produced or the magnetic field is about to be ramped up, that the environment is safe for all people working or spending any time adjacent to the unit. This work is covered by acceptance testing which is then followed by extensive Linac commissioning tasks and additional MRI specific testing.

The MRI-Linac is not a standalone device but is integrated with multiple other major systems with a health service. In parallel to the machine commissioning tasks, the ROMPs will establish networked communication between all major equipment supporting the MRI-Linac including the Oncology Information Management Systems, the radiotherapy treatment planning systems, and other major IT services within the oncology department. End-to-end data integrity checks will be undertaken to ensure data integrity is maintained; electronic medical record storage will be established; quality assurance processes will be created, and all documentation and credentialing standard operating procedures will be written and ratified by peer review and independent testing prior to patient treatment commencing. Systematic error trapping will also be established, and prospective data capture systems will be put in place to support prospective research and clinical studies.

ROMPs are responsible for the accuracy and range of clinical applicability of the treatment planning systems. Conditions of irradiation encountered with the MRI-Linac, differ considerably from conventional Linacs. Extensive testing of all aspects of the treatment planning system, especially including new features such as plan adaption routines, will be needed before clinical use. The quality and spatial integrity of patient-specific data such MRI-Linac acquired images used for treatment planning requires ROMP evaluation during the commissioning phase.

All equipment specific to the MRI-Linac must be commissioned for MRI compatibility and modelling within the radiation planning system. It is likely that this equipment, and the patients on which it will be used, will also undergo CT (Computed Tomography) scanning and so a similar set of tests must be conducted for CT commissioning. ROMPs are responsible for testing and authorizing the use of such equipment for its inclusion in clinical protocols.

The commissioning of an MRI-Linac will be accompanied in many clinics by pre-requisite technologies for dose adaption and re-thinking of process steps. Clinical steps such as simulation, treatment planning, image fusion, motion management, dose accumulation, plan approval are all topics that need to be addressed by the ROMP workforce.

### Routine physics activities

#### Patient treatment provision

Simulation, planning, motion management, and treatment protocols for tumour streams will be developed by multidisciplinary clinical teams (Radiation Oncologists, Medical Physicists, Radiographers Radiation Therapists and Nurses). Individual patient treatment will be reviewed for quality and accuracy by an MRI-Linac team including ROMPs. Adapting plans and developing efficient, real-time QA processes unique to the MRI-Linac also require a ROMP to be available for patient planning and treatment.

Patients will receive numerous scans prior to treatment. ROMPs will undertake benchmarking and generate metrics to quantify variation in image quality between simulation and treatment imaging systems. The complexity and variation between source simulation MRI images and treatment MRI images can vary over time and must be monitored and accounted for within the treatment process. Translational work migrating new modalities and planning strategies, such as synthetic CT or the adoption and application of functional imaging into clinical practice are examples of work that is routinely progressed from novel to the release for incorporation into routine clinical practice by ROMPs.

#### Quality assurance and maintenance of equipment

MRI-Linacs, in common with conventional Linacs, require substantial, frequent effort to verify and maintain constancy, quality and spatial precision of dose delivered to patients undergoing treatment. The Medical Physicists will be responsible for developing and managing quality assurance testing programs specific to MRI-Linacs. ROMPs will apply all existing guidelines and legislation to the ongoing QA and maintenance of the MRI-Linac and subsidiary major equipment. Where none exists specific to MRI-Linacs, they will review both radiotherapy and magnetic resonance image guidance publications and adapt them to the MRI-Linac environment. They will manage documentation, such as authorship of SOPs (Standard Operating Procedures), recording and review of test results, and education of other staff as needed to undertake QA tasks. ROMPs will have overarching responsibility for the performance of the MRI-Linac.

### Radiation and MR safety & quality management

#### Procedures and policies

An MRI-Linac, with innovative technology and new protocols, requires development of complete, mostly uncharted components of quality checks throughout planning and treatment systems. To migrate knowledge specific to local health service circumstances ROMPs should be tasked at the outset of scoping and acquisition of MRI-Linac service development to contemporaneously develop documentation to support policy development (whether safety, operational or clinically relevant) for the commencement of clinical service.

ROMPs understanding of MRI-Linac engineering design, radiation delivery, patient physiology within an MRI setting, and data flow throughout the treatment chain; along with an intrinsic understanding of how radiation behaves in an MRI field and how this is modelled by the treatment planning system is the basis for advising on safe conditions and use of MRI-Linacs.

Training, education, and application for treatment is substantially more technical than current practice. These elements require the development of a robust credentialing program and documentation for a broad range of radiotherapy staff.

#### MRI and radiation safety

Medical Physicists are responsible for ensuring the MRI-Linac is safe to use from a radiation shielding perspective, an MRI electromagnetic perspective and a fit-for-purpose perspective; this review of both the Radiation and MRI elements translate to ensuring it is safe to treat patients.

Medical Physicists will need to create departmental specific safety guidelines considering safety issues for both the MRI and Linac. The Medical Physicist will have or should undertake MRI Safety certification [15–17] which will combine with their existing radiation certification [18] to provide expert advice to the radiotherapy department in the delivery of patient treatment. ROMPs will work with colleagues to develop the program and supporting materials as well as the creation of a practical induction program. This will be run frequently; before go live, and throughout the clinical life of the MRI-Linac. A robust incident review system aligned with local clinic practice will heavily involve ROMP responsibility under the remit of the MRSE or MRSO and RSO roles [6].

Adherence to state and national guidelines and legislation applies across all phases of the purchase, installation, and use of the MRI-Linac. Guidelines and legislation are living documents, the Medical Physicists’ extent of professional operation incorporates maintenance of currency with all relevant regulations and legislation as it applies to the context of clinical practice. Where no Australian or New Zealand guidelines exist, or the legislation is silent, it is the responsibility of the Medical Physicists to review international documentation and adapt it for local circumstance in consultation with appropriate professional bodies or colleagues. Documentation and due diligence shall be maintained by ROMPs in this regard.

#### Education and training

Medical Physicists are experienced educators, having familiarity with current radiotherapy practice and quality processes combined with expertise in radiation safety making them ideally suited to implement MRI safety, education, and credentialing across the broad radiotherapy workforce. Development of induction procedures, credentialing programs and providing ongoing CPD (Continuing Professional Development) will contribute to the maintenance of high levels of safety required in the workplace.

### Research in radiotherapy; clinical advancement and translation

#### Research and development

There is an immediate and ongoing demand for research and development in the field of MRI in radiation oncology. It is currently a new clinical service and is undergoing rapid expansion; this expansion will continue for the foreseeable future. Research and mapping of MRI/RT from concept to prototype/beta testing and subsequent clinical validation, is best met by the involvement of ROMPs attached to clinical services. With a high level of scientific understanding of the concepts and techniques used to scan, plan and deliver radiation treatment to patients in combination with MRI imaging, Medical Physicists’ research activity will efficiently and safely expedite improvement in treatment options, patient outcomes and provide upstream knowledge to vendors for development of clinically beneficial workflows and supports. Medical Physicists’ strong scientific training allows them to collaborate with existing physics lead researchers on MRI in radiotherapy and with international colleagues and vendors to interpret and adapt development for local environments. They have the appropriate skills to disseminate information and provide education for clinical and research driven activities within services. Finally, they are very well placed to provide supervision and support for other researchers within clinical departments.

### Data, IT & engineering management & support

#### First-line maintenance

ROMPs should be present as much as possible during machine installation and setting to work undertaken by the vendor engineers and installers. This is a unique event in the life of any linac and is a prime opportunity for ROMPs to gain intrinsic knowledge of the MRI-Linac for which they will be responsible when the move to treatment of patients occurs. It is a prime opportunity to gain first-hand knowledge and to establish rapport with the vendor engineers which will translate to improved communication to support the service. First-line maintenance training is most beneficial when conducted adjacent to machine installation. ROMP understanding of engineering, field production, radiation, and electromagnetic interplay can be consolidated through practical first-line maintenance training. When services go live the presence of a competent ROMP to troubleshoot MRI-Linac issues and communicate at a technical level with engineers will improve machine reliability and will enable a safe, efficient service to be maintained. Where feasible, it is recommended that ROMPs undertake first-line training.

#### Network and data management

The various apparatus used in radiation oncology do not exist in isolation, but rather as a complex system with varying levels of dependency. Critical to the safe delivery of radiation treatments is the management of data storage, manipulation, and transfer which underpin the operations of a modern radiation oncology facility. ROMPs have a shared responsibility with other departmental staff groups to ensure these elements are managed appropriately. With specialised training in the data types used and applications within the clinic, ROMPs are ideally placed to fulfil these responsibilities.

## Conclusion

The scope of practice of the ROMP, as pertaining to the MRI-Linac, in the Australian and New Zealand context, has been elaborated. Safety must be addressed considering the particular risk profile of the MRI-Linac and ROMP engagement, throughout all phases of the machine lifecycle, is crucial to safety. Emphasis has been placed on early involvement and recruitment of the ROMP workforce. Staffing levels should be determined by applying the guidance given in this position paper to the ACPSEM ROMP workforce model using the accompanying calculator. Example MRI-Linac specific inputs have been given as an illustration.

## Supplementary Information

Below is the link to the electronic supplementary material.

Supplementary file1 (PPTX 949 KB)
