# Supplementary figures and images for: ACPSEM position paper on ROMP scope of practice and staffing levels for magnetic resonance linear accelerators
Source: Phys Eng Sci Med. 2023 Apr 11;46(2):521–7. doi: 10.1007/s13246-023-01253-4 (PMC10209343; doi:10.1007/s13246-023-01253-4)

## Slide 1
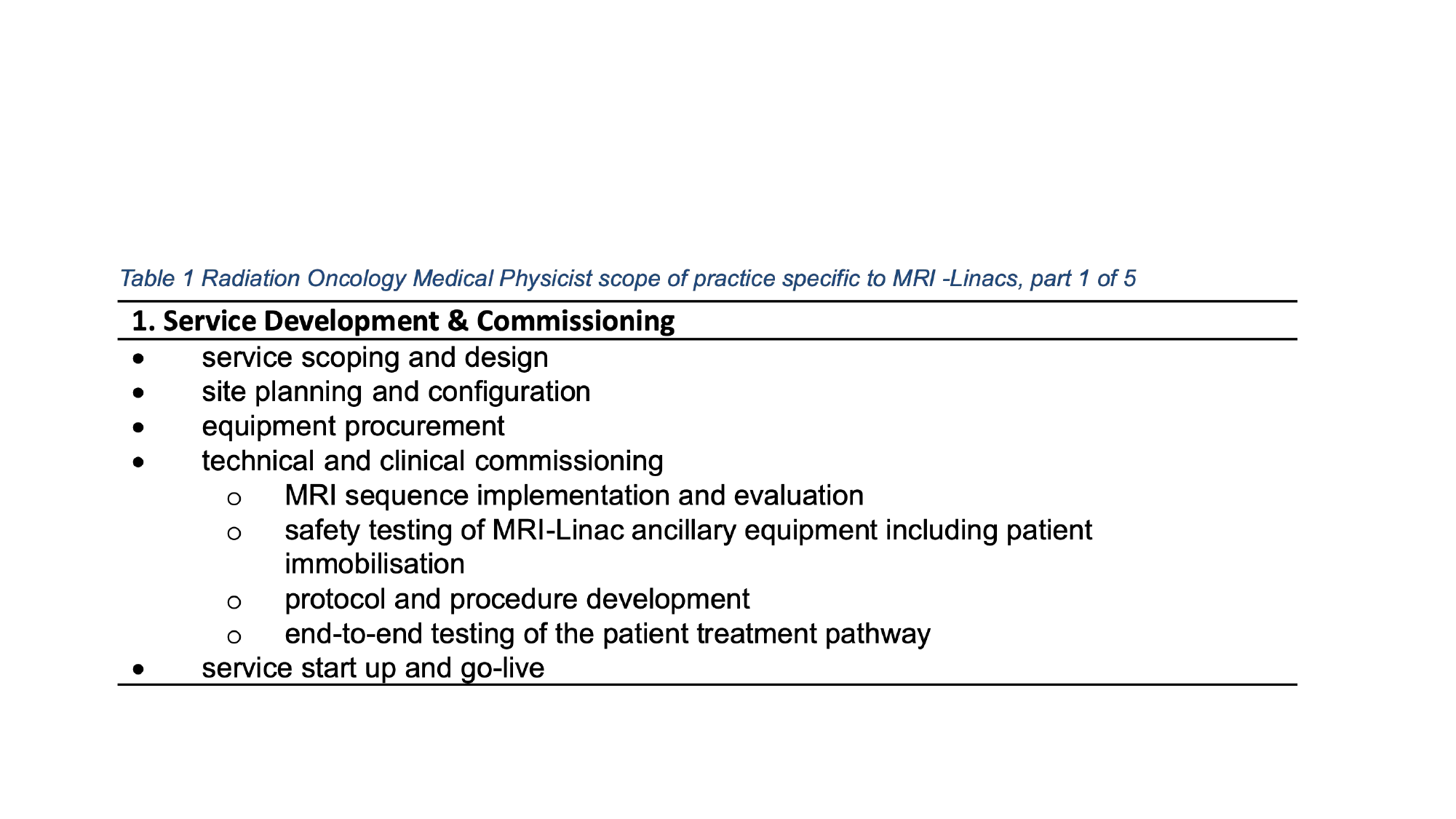

## Slide 2
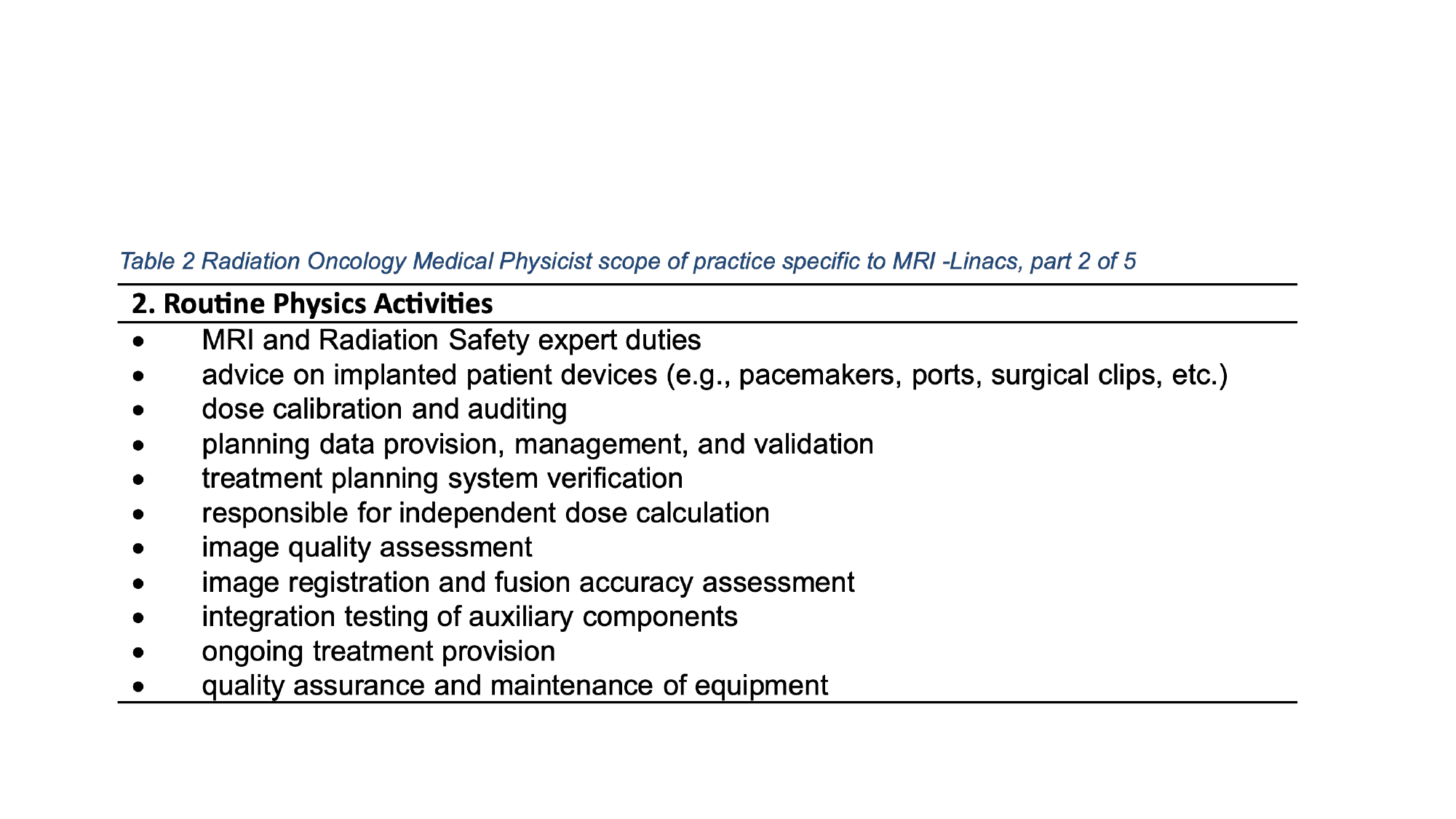

## Slide 3
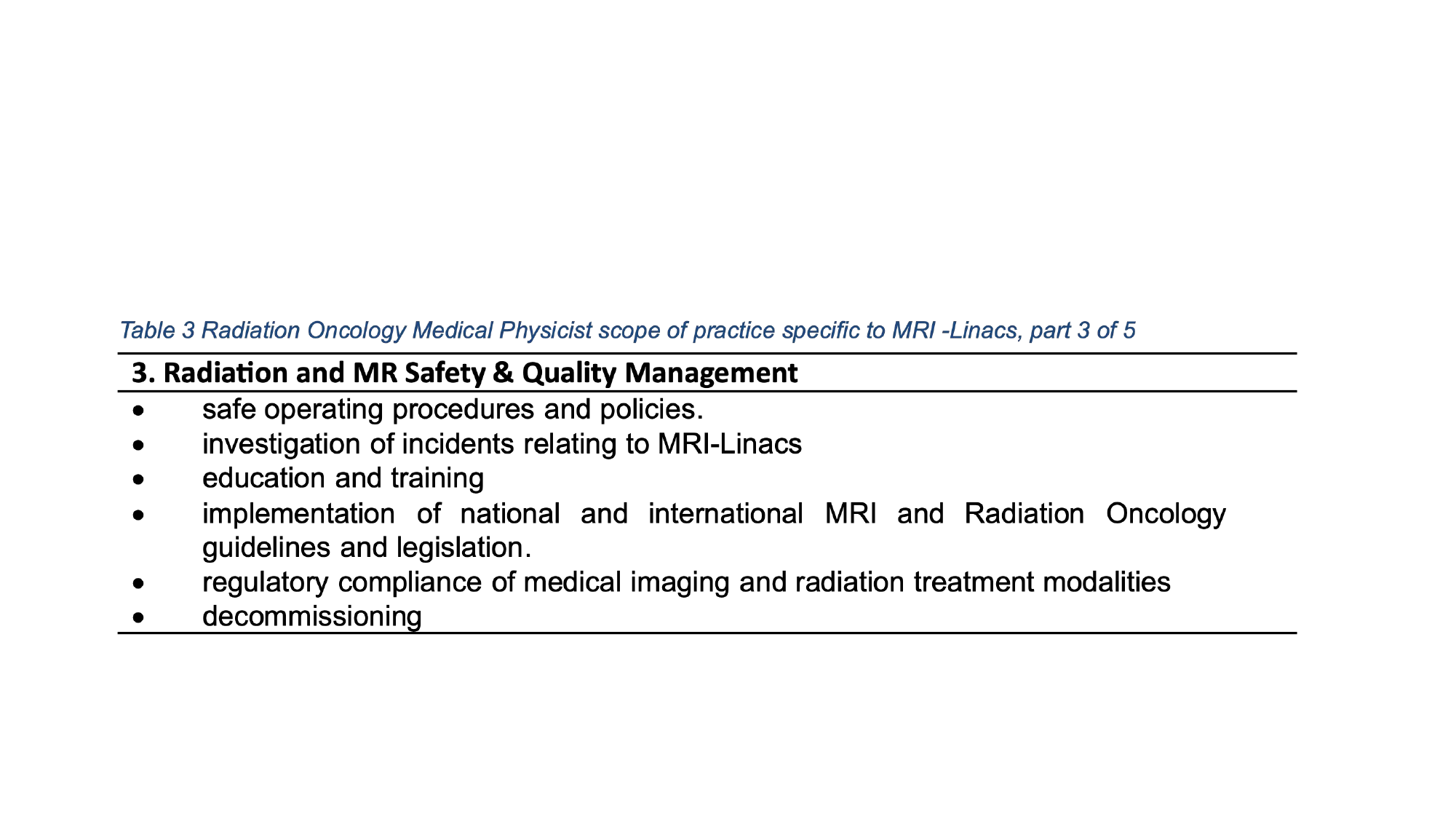

## Slide 4
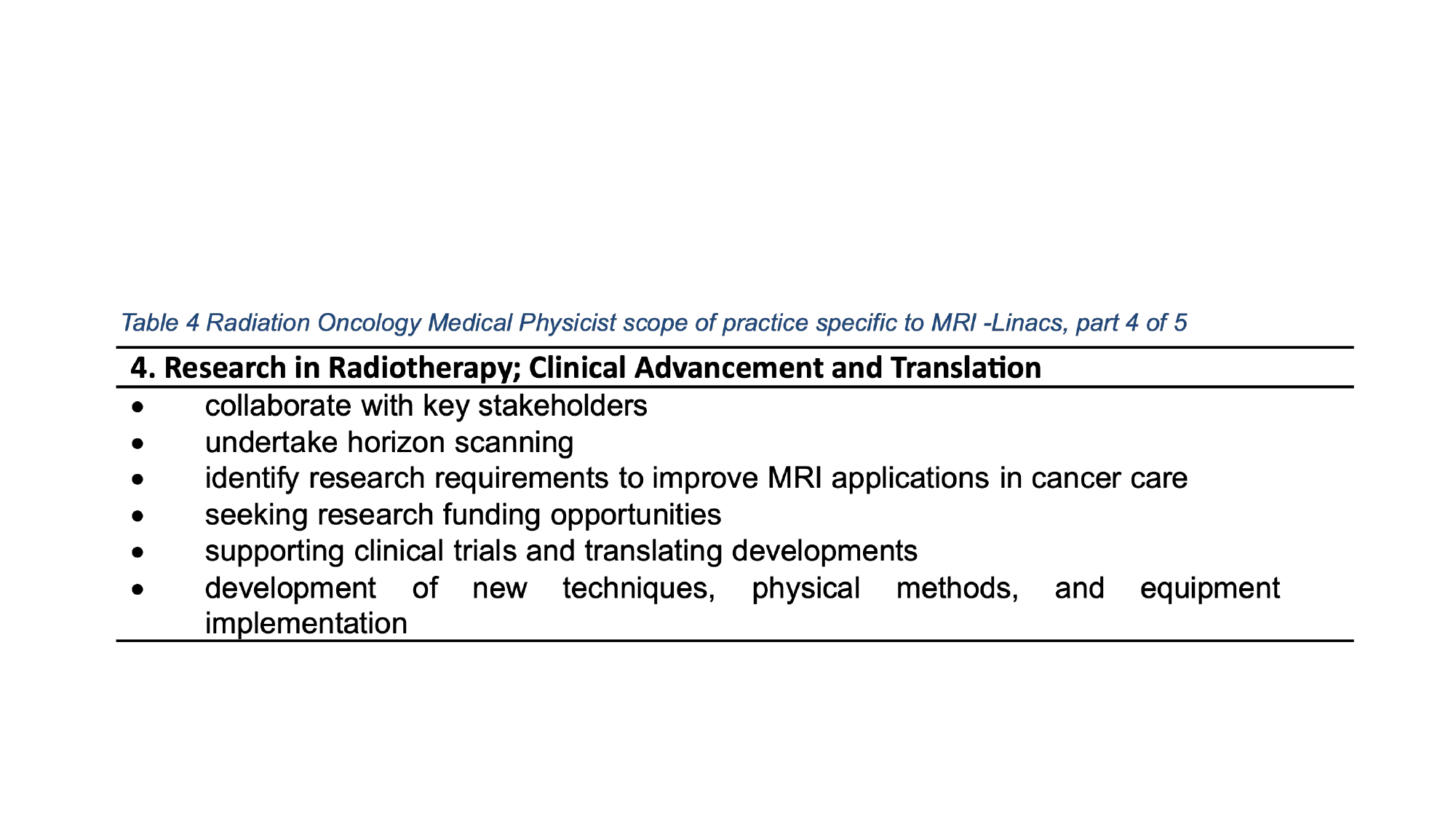

## Slide 5
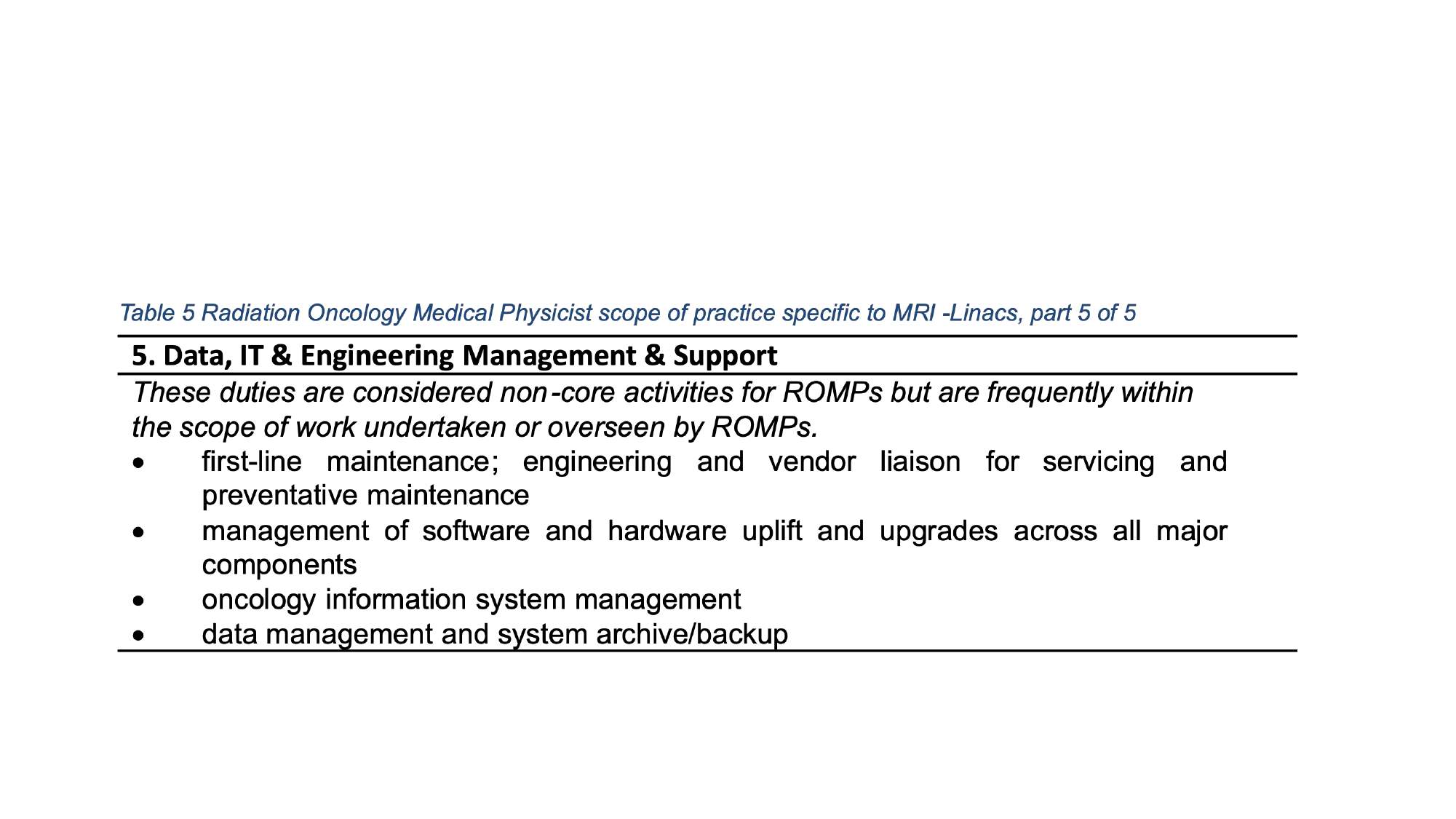

Supplement: Supplementary file 1 — Supplementary file1 (PPTX 949 KB) [file 13246_2023_1253_MOESM1_ESM.pptx]
